# Research advances in Zein-based nano-delivery systems

**DOI:** 10.3389/fnut.2024.1379982

**Published:** 2024-05-09

**Authors:** Xiaoxuan Liu, Minhong Zhang, Xuelian Zhou, Mengjiao Wan, Aiping Cui, Bang Xiao, Jianqiong Yang, Hai Liu

**Affiliations:** ^1^College of Pharmacy, Gannan Medical University, Ganzhou, China; ^2^Department of Clinical Medicine Research Center, The First Affiliated Hospital of Gannan Medical University, Ganzhou, China; ^3^Ganzhou Key Laboratory of Antitumor Effects of Natural Products, Ganzhou, China

**Keywords:** Zein, nano-delivery system, nanoparticles, self-assembly, active targeting, intelligent response nanoparticles

## Abstract

Zein is the main vegetable protein from maize. In recent years, Zein has been widely used in pharmaceutical, agriculture, food, environmental protection, and other fields because it has excellent biocompatibility and biosafety. However, there is still a lack of systematic review and research on Zein-based nano-delivery systems. This paper systematically reviews preparation and modification methods of Zein-based nano-delivery systems, based on the basic properties of Zein. It discusses the preparation of Zein nanoparticles and the influencing factors in detail, as well as analyzing the advantages and disadvantages of different preparation methods and summarizing modification methods of Zein nanoparticles. This study provides a new idea for the research of Zein-based nano-delivery system and promotes its application.

## Graphical abstract

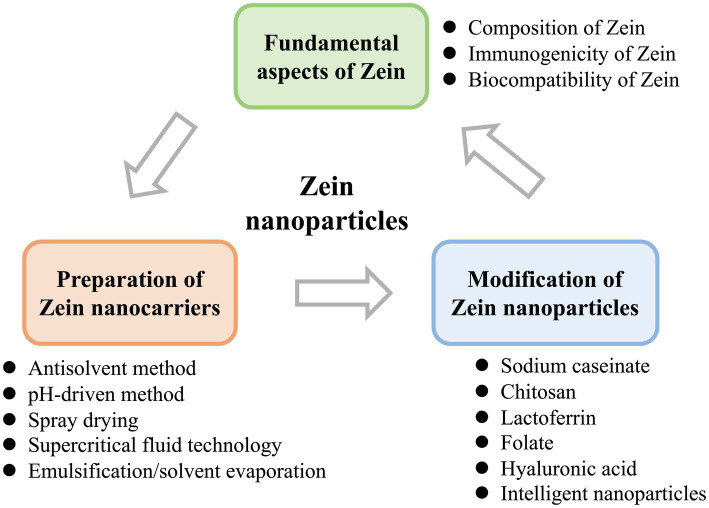

## 1 Introduction

The low solubility and poor stability of drugs have become the obstacles to their current application (1–4). In recent years, advanced materials such as micelles, liposomes, proteins, and metal nanomaterials have obvious advantages in maintaining drug stability, prolonging blood circulation time, controlling drug release, improving bioavailability, reducing toxicity, and enhancing cell absorption (5–9). Natural protein is promising alternatives material to traditional synthetic materials due to environment-friendliness (10, 11). Besides, compared with animal protein, plant protein has received a lot of attention from researchers due to their advantages of inexpensiveness and ready accessibility (12).

Maize is considered the third most important cereal in the world (13). Zein is a kind of natural plant protein extracted from maize and is the second most important nutrient in maize (14, 15). Zein lacks lysine and tryptophan, two essential amino acids for humans, which means that maize kernels contain relatively poor-quality protein as food (16). However, Zein has the characteristics of ideal drug delivery carrier such as self-assembly, low immunogenicity, good biocompatibility, and easily modifiable (12, 17). Based on the long history of Zein usage in food and related evaluation data, Zein has been used as an ideal material for food technology and drug delivery since 1939. In 1985, Zein had been granted Generally Recognized As Safe (GRAS) status by US-FDA (5, 18). In recent years, the research on Zein-based nano-delivery system has covered many fields such as food nutrition (19, 20), drug delivery (5, 21–25), agriculture (26), and environmental protection (27, 28). In addition, Zein as a carrier material has been designed into nanoparticles, nanocapsule, films, and nanofibres. However, the systematic summary and comprehensive review of Zein-based nano-delivery system is still lacking.

In first part, the composition, immunogenicity, biocompatibility, and other basic properties of Zein were summarized. Then, the principles, influencing factors, advantages and disadvantages of different preparation methods of Zein nanoparticles are discussed. Finally, the optimization effect of different modification methods on the function of Zein nanoparticles was researched (Graphical Abstract). The purpose of this paper is to summarize and discuss the research of Zein-based nano-delivery system in recent years, and to lay a foundation for further research of Zein-based nano-delivery system and provide a new research idea.

## 2 Fundamental aspects of Zein

### 2.1 Composition of Zein

Zein is a prolamin protein isolated from the endosperm of maize and makes up about 80% of the entire protein in maize (23). Zein is rich in hydrophobic and neutral amino acids (such as leucine 20%, proline 10%, and alanine 10%), but lacks lysine and tryptophan. Furthermore, the small number of arginine and histidine residues in the structure of Zein are the main differences between Zein and other proteins (5). The fixed amino acid composition provides Zein with unique solubility (25). Therefore, Zein is insoluble in water, but soluble in alkaline solutions (pH ≥ 11.5), 70–95% aqueous ethanol solutions and water-acetone solutions (17).

Zein is classified into four categories (α-Zein, β-Zein, γ-Zein, and δ-Zein) based on their different conformational arrangements, molecular sizes, molecular weights, and solubilities (12). The specific proportions and molecular weights of the four Zein species are shown in Table 1. The primary structure and secondary structure of Zein can be determined by chromatography, circular dichroism, Fourier Transform Infrared Spectroscopy (FTIR) and Nuclear Magnetic Resonance (NMR) (29–31). At present, studies on the structure of Zein mainly focus on α-Zein and γ-Zein. The α-Zein consists of highly homologous repeating units and has a high α-helix content of 35–60% (32, 33). Several different models of the α-Zein tertiary structure have been proposed: cylindrical model, ribbon model, hairpin model, and super helix model (5, 23, 34). In addition, the secondary and tertiary structures of α-Zein were affected by different solvent types, for example, Daniel et al. showed that the α-helix structure in α-Zein decreased with the increase of water content in the ethanol-water system, and the β-sheet content increased with the increase of the proportion of hydrophilic solvent (35). Research by Wang and Padua also supports this argument (36, 37). Moreover, both α-Zein and γ-Zein are rich in glutamine, alanine, leucine, and proline, but differ in their cysteine content. Specifically, α-Zein contains 1–3 cysteine residues, while γ-Zein contains 12–15 cysteine residues, which can form disulfide bonds to stabilize protein structures (38, 39). The glutamine-rich structure of γ-Zein with molecular weight of 27 kDa may be involved in protein interaction and protein oligomerization. The N-terminal repeat domain (VHLPPP) of γ-Zein is related to membrane permeability and has the ability to cross cell membranes, which allows it to use as a carrier peptide to facilitate drug absorption (40–42).

**Table 1:** Weight and proportion of four kinds of Zein.

Kind	Molecular weight	Proportion
α-Zein	19 and 22 kDa	75–85%
β-Zein	14 kDa	10–15%
γ-Zein	16 and 27 kDa	5–10%
δ-Zein	10 kDa	

### 2.2 Immunogenicity of Zein

Immunogenicity is a key factor to be considered in the preparation of drug delivery systems *in vivo*. Compared with animal protein, Zein is more readily available and has less immunogenic potential (43). Certainly, the route of administration affects antigenicity of Zein (5). Through mice experiments, Pepi et al. proved that intramuscular injection of Zein triggers a systemic immune response. The oral administration of Zein nanoparticles did not cause systemic immune response but induced systemic tolerance without mucosal tolerance (44). Park et al. (45) showed that inhaling Zein dust could induce type 1 hypersensitivity reaction leading to asthma. Zein is hydrolyzed by gastrointestinal proteases and still causes allergic reactions in celiac patients, which is related to IgA antibodies in celiac disease patients recognizing digested α-Zein as celiac disease antigens (46). The 50 kDa protein in maize is a reduced soluble protein as defined by Wilson et al. and this protein has been identified as potential allergen for maize allergy sufferers by Pasini’s study. Moreover, Lee et al. identified the 50 kDa protein in maize as 50 kDa γ-Zein (47–49). Immunological experiments in mice showed that Zein nanoparticles with particle size between 100 and 400 nm had no immune response, but the particle size greater than 400 nm can lead to an immune response two to four times higher than the saline group (50). In addition, the cells involved in nanoparticle-triggered immune responses are mainly phagocytes (51). Easier adsorption of proteins on the surface of hydrophobic nanoparticles leads to their easier uptake by phagocytes (52). The immune response induced by Zein may be related to hydrophobic amino acids such as glutamine, leucine and alanine in Zein. Therefore, the immunogenicity of Zein nanoparticles was related to the route of administration, particle size, and hydrophobicity.

### 2.3 Biocompatibility of Zein

Biocompatibility was redefined in 1987 as the ability of a material to perform with an appropriate host response in a specific situation (53). Materials with excellent biocompatibility should satisfy the requirements of non-toxicity, non-immunogenicity, non-thrombogenicity, and non-carcinogenicity of themselves and their metabolites (54–56). The safety of biological materials is crucial for drug delivery, so biocompatibility is a necessary area in the material research process (57). In the past few years, due to the biocompatibility and degradability of Zein, the application of Zein polymer in drug delivery and tissue engineering has been widely studied (58). Dong et al. investigated the effects of Zein on the attachment, morphology, and proliferation of HL-7702 cells and NIH3T3 cells by microscopic observation and MTT experiment, and the results showed that Zein film with low concentration and small particles had better cell proliferation ability, which proved that Zein had good biocompatibility (59). Liu et al. (60) showed that Zein-fucoidan complex nanoparticles had good biocompatibility. In addition, hemolysis test and cell culture experiments demonstrated that Zein had no hemolysis effect and low cytotoxicity (61) Studies by Gong (62), Wang (63), and Kim (64) also prove this view.

## 3 Preparation of Zein nanocarriers

### 3.1 Antisolvent method

**Figure 1:**
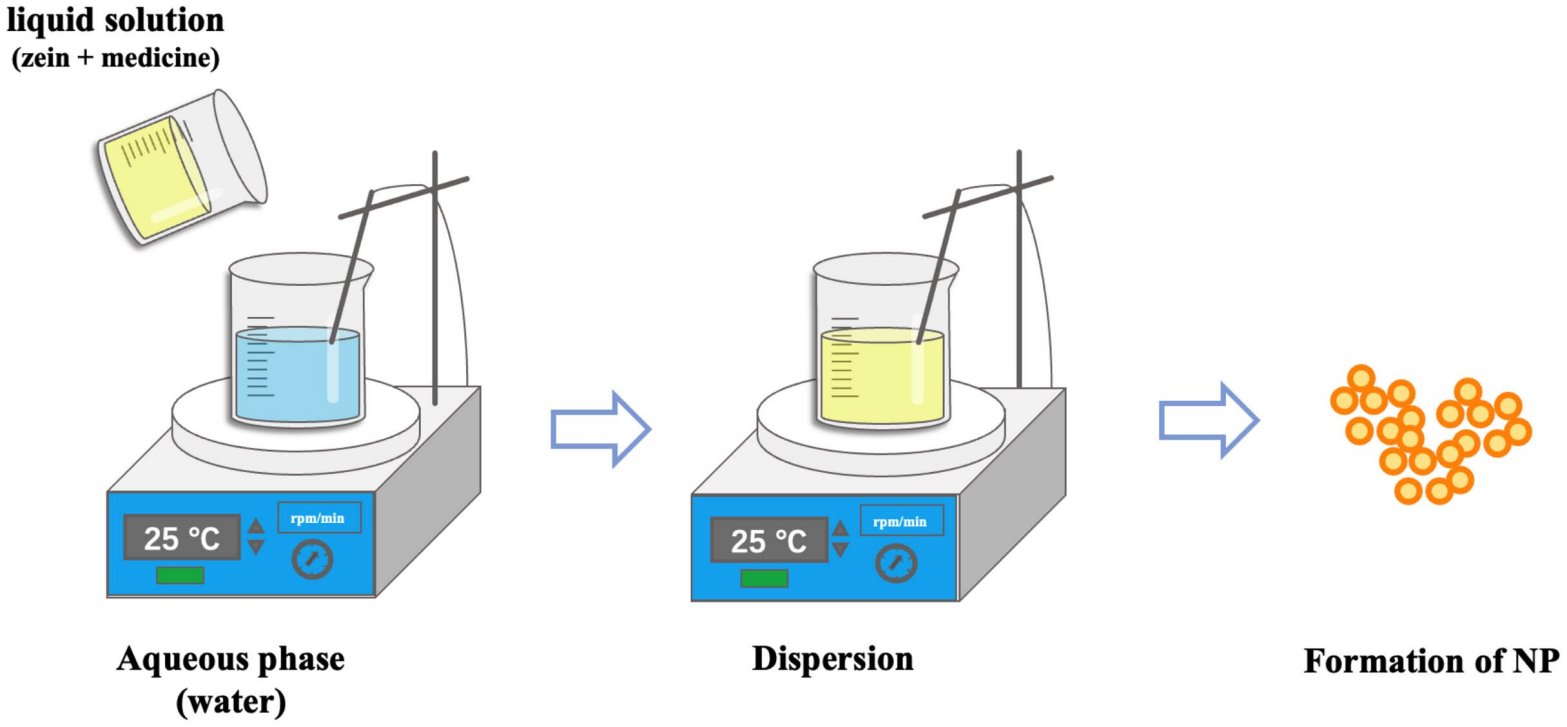
Preparation of Zein nanoparticles by Antisolvent methods.

Antisolvent precipitation, also known as phase separation or liquid–liquid dispersion, is a common preparation method for Zein nanoparticles (65). Deionized water is usually used as the antisolvent, and ethanol aqueous solution is used as the solvent of Zein (66). The basic principle of this method is to prepare nanoparticles by changing the polarity of the solvent around Zein. In this process, the proportion of organic solvent is reduced, the solubility of Zein is reduced, and the nanoparticles are self-assembled (67, 68). The unique solubility of Zein makes it to self-assemble into nucleus-like particles in the antisolvent and the nucleus can grow further by trapping non-aggregated solute molecules, but the particle growth will stop when the concentration of Zein in the solvent is too low (69–71). The preparation method is shown in Figure 1. Lou et al. prepared Zein nanoparticles with a particle size of 252.8 ± 7.3 nm by dissolving Zein in 70% aqueous alcohol solution and injecting anti-solvent under vigorous stirring. The particle size of the nanoparticles prepared by adding the stabilizer carboxymethyl chitosan was 113 nm, which is a decrease in particle size compared to the nanoparticles without stabilize (72). Ye et al. dissolved Zein in 80% ethanol solution and added it into water at a rotational speed of 1,200 rpm to prepare nanoparticles with a particle size of 209.2 ± 1.9 nm and Zeta-potentials of-19.0 ± 0.7 mV (73). Lonare et al. (74) found that polymer concentration was negatively correlated with nanoparticle size, and the higher the polymer solution concentration was, the smaller the nanoparticle size was obtained. Therefore, when nanoparticles were prepared by antisolvent methods, the polymer content, the type of solvent and non-solvent, the ratio of solvent to non-solvent, the addition rate of solvent to non-solvent, the action of stabilizer, and the stirring speed all affected the particle size of the nanoparticles.

### 3.2 pH-driven method

**Figure 2:**
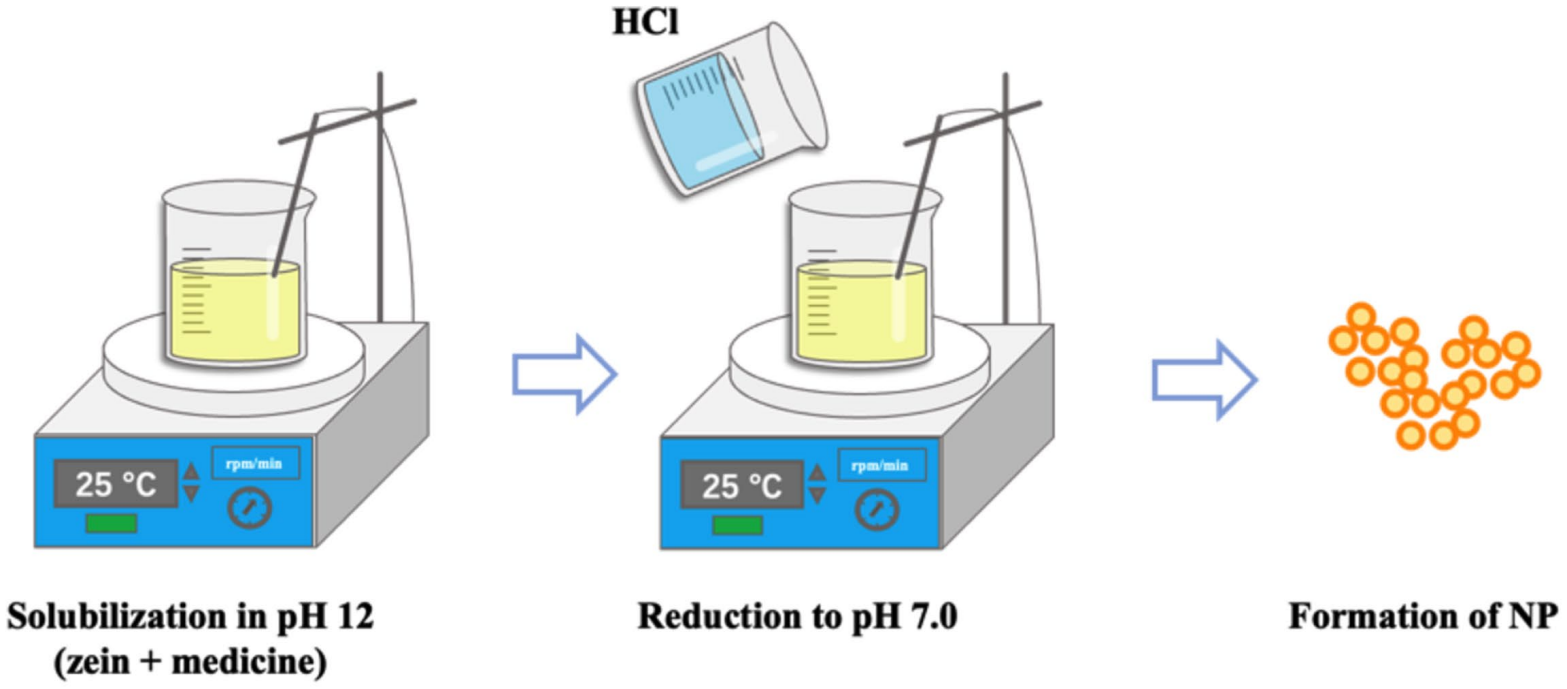
Preparation of Zein nanoparticles by pH-driven methods.

The preparation of Zein nanoparticles by antisolvent method is simple and efficient, but it may bring some safety risks due to the large amount of organic solvents involved (75). The principle of the pH-driven method is based on that Zein is soluble in alkaline solutions and insoluble in neutral or acidic solutions. Therefore, Zein and the drug can be dissolved in deionized water under magnetic agitation at pH 12.0, then NaOH and HCl are used to adjust the pH of the deionized water. Finally, Zein nanoparticles were formed when the pH of the solution changed from alkaline to neutral under agitation (76). The preparation method is shown in Figure 2. At present, the pH-driven method has been widely used to prepare Zein nanoparticles (77). For example, the natamycin-loaded Zein-casein nanoparticles (N-Z/C NPs) were prepared by pH driven method. The average particle size of the nanoparticles was <100 nm and the Zeta potential < −30 mV (78); Yuan et al. prepared Zein/Tea saponin composite nanoparticles (Z/TSNPs) using pH-driven method. Zein and Tea saponin (TS) were dissolved at pH 12.0, after which the above solution was adjusted to pH 7.0 using HCl, and Zein nanoparticles were obtained by centrifugation at 2,000 g for 10 min, with encapsulation efficiency of 83.73% and loading capacity of 22.33%. Besides, Z/TSNPs increased the solubility of curcumin by about 290 times (79). However, nanoparticles prepared by the pH-driven method tend to aggregate and even form irregular precipitates (80). In practical applications, it is not only necessary to select drugs and carrier materials whose solubility varies with pH, but also to consider whether high-alkali solutions will cause drug degradation (81, 82).

### 3.3 Spray drying

**Figure 3:**
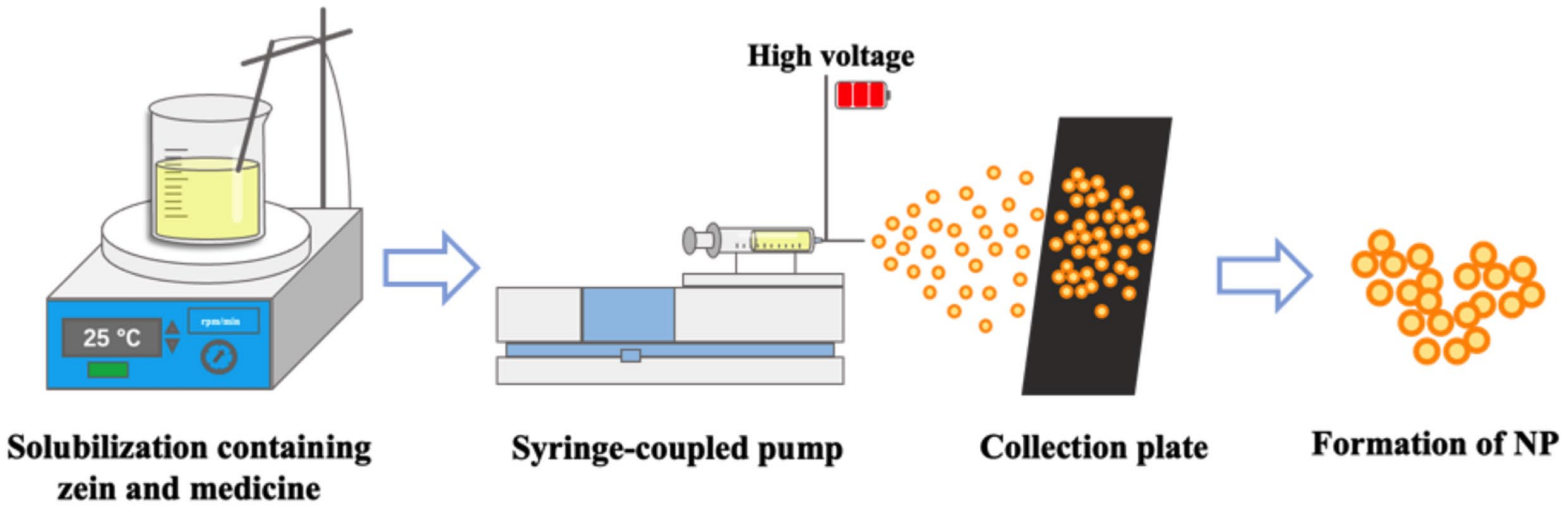
Preparation of Zein nanoparticles by Spray drying.

Spray drying, also known as electrohydrodynamic atomization, is a frequently used technology for producing dry powder in pharmaceutical industry (83). The four key procedure of spray drying are atomization, droplet–to–drying gas contact, particle drying, and particle collection (84). The method mainly depends on spraying the material to be dried in hot air by mechanical action, the solvent is evaporated, and the resulting ultrafine powder is collected (85). The preparation method is shown in The preparation method is shown in Figure 3. According to the literatures, the solubility of the drug and polymer, the choice of solvent, and the temperature affect the prepared nanoparticles. Especially the increase of temperature can increase the solubility of the drug and polymer, and make the prepared nanoparticles more homogeneous (86–88). Zein based nanoparticles can also be produced by spray drying liquids containing already-formed Zein nanoparticles or by spray drying an ethanol aqueous solution of Zein. Francisco et al. dissolved Zein in 80% ethanol solution and prepared Zein nanoparticles loaded with quercetin by spray drying method. The parameters of the equipment used for the preparation of nanoparticles: electrical potential of 15 kV; flow rate of 0.1 mL/h; and distance from the needle to the collector of 15 cm. The encapsulation efficiency of quercetin in the prepared Zein nanoparticles reached 87.9 ± 1.5 to 93.0 ± 2.6%, and the 4 h release of quercetin in the simulated experiment of gastrointestinal tract was 79.1% (89). Baspinar et al. prepared Zein nanoparticles loaded with curcumin by spray drying method, with a particle size of about 500 nm (90, 91). However, the high temperatures during production of this method may have an impact on drug stability, and the technique should be used with caution when preparing drug delivery systems loaded with heat-sensitive compounds (92, 93).

### 3.4 Supercritical fluid technology

**Figure 4:**
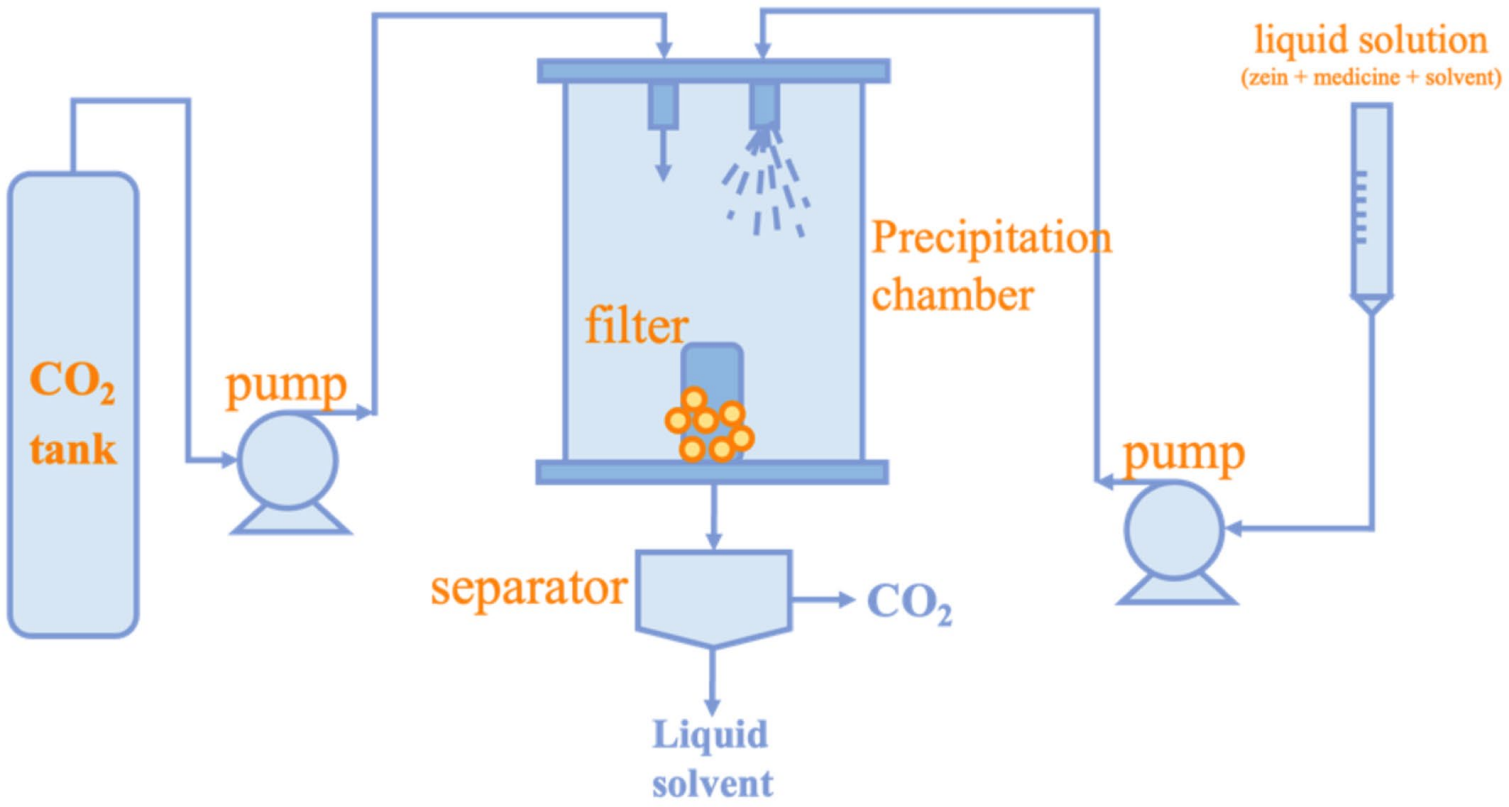
Preparation of Zein nanoparticles by Supercritical fluid technology.

The supercritical fluid (SCF) technology is an efficient and environmentally friendly method for preparing nanoparticles (94). The simple process of supercritical fluid technology is to extract the co-solvent in the atomized droplet by supercritical CO_2_ after continuous injection of the feedstock. Since most polymers are insoluble in CO_2_, the solubility of the polymer gradually decreases, thus forming atomic nuclei and gradually growing into nanoparticles or microparticles (95). The preparation method is shown in Figure 4. Compared with other preparation methods, supercritical fluid technology can effectively remove organic solvents and is friendly to heat-sensitive bioactive substances so that they do not degrade during particle formation (96). In recent years, with the improvement of science and technology, supercritical fluid technology can produce smaller and more controllable particles (97). The main factors that affect particle size when nanoparticles are prepared using SCF include temperature, pressure, properties of organic solvent, solute concentration, anti-solvent and solution flow rate, and the geometry of the chamber and nozzle. Hu et al. used this technique to prepare Lutein/Zein nanoparticles with a particle size of approximately 200 nm at a pressure of 10 MPa, the Lutein/Zein ratio of 1: 18 (w/w), the solution flow rate of 1.0 mL/min, and the temperature of 45°C. And it was demonstrated that lower temperatures and solution flow rates coupled with high pressures favored smaller, more regular nanoparticles (98). Li et al. successfully prepared Zein nanoparticles with a minimum size of 50 nm by SCF. The concentration of Zein was fixed at 10 mg/mL, and it was dissolved in a mixture of ethanol and dichloromethane at a volume ratio of 5: 7. The specific operating parameters of the apparatus were: temperature of 45°C; pressure of 10 MPa; and flow rate of the dosing solution of 1 mL/min. The study showed that the nozzle structure and CO_2_ flow rate affected the morphology and size of the nanoparticles (99).

### 3.5 Emulsification/solvent evaporation

**Figure 5:**
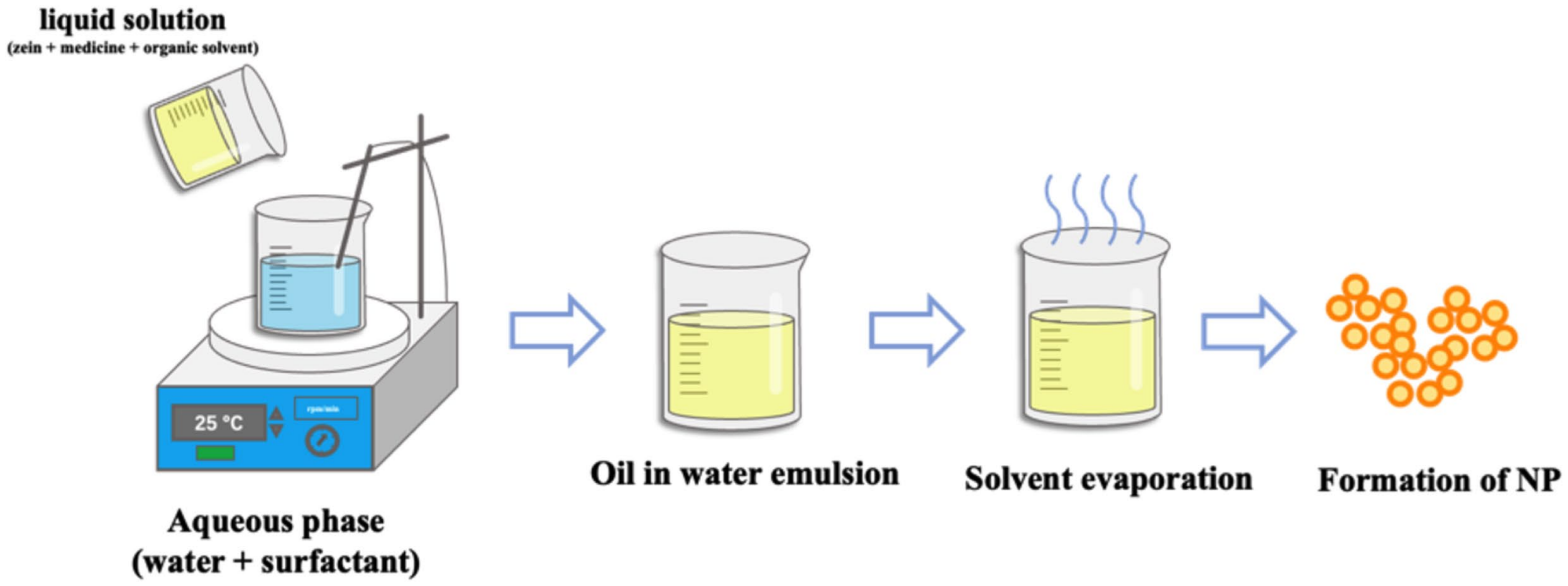
Preparation of Zein nanoparticles by Emulsification/solvent evaporation.

The preparation of nanoparticles by emulsification/solvent evaporation technology is mainly divided into two parts, the first step is to emulsify the polymer solution into an aqueous phase, and the second step is to evaporate the solvent. Specifically, the emulsification/solvent evaporation is the process of dissolving selected polymers in a volatile organic solvent (oil phase), and then injecting the oil-phase solution into an aqueous solution containing a surfactant to obtain a nanoemulsion. Upon evaporation of the organic solvent, drug-loaded nanoparticles in the aqueous surfactant solution are obtained (100–102). Therefore, the diameter of nanoparticles can be controlled by adjusting the stirring speed, the viscosity of aqueous phase and organic phase, the type and concentration of dispersing agent when emulsified solvent evaporation is used to prepare nanoparticles (103). The preparation method is shown in The preparation method is shown in Figure 5. Yang et al. prepared Zein nanoparticles loaded with resveratrol by emulsification/solvent evaporation method. The particle size of the nanoparticles was 389.90 ± 3.02 nm, the Zeta potential was −13.37 ± 0.26 mV, the encapsulation efficiency was 64.17 ± 0.07%, and the drug loading was 5.78 ± 0.01% (104).

The advantages and disadvantages of different preparation methods are summarized in Table 2.

**Table 2:** Advantages and disadvantages of different preparation methods.

Method	Advantages	Disadvantages
Antisolvent methods	Low cost	The size of np is affected by many factors
	Simple operation	Limited scalability
	Does not require heating	The size of np is affected by many factors
pH-driven method	Simple operation	Drug degradation
	Does not require heating	Nanoparticle aggregation
Spray drying	Easy scalability	Collection difficulty
	Simple operation	Low production efficiency
	High encapsulation efficiency	Requires heating
Supercritical fluid technology	Safety and green process	High equipment requirements
	High process yields	High maintenance cost
	Low process complexity	
Emulsification/solvent evaporation	Simple operation	Residual solvent
	Easy scalability	Requires heating for evaporation
	Stable properties of nanoparticles	Lower efficiency in water-soluble drugs encapsulation

## 4 Modification of Zein nanoparticles

### 4.1 Sodium caseinate

Casein is the main protein of milk in the form of macromolecular aggregates (105, 106). Sodium tyrosine is a commonly used nutrient and functional component in emulsion preparation due to its good solubility and emulsification properties (107). Caseinate moves the isoelectric point of the colloidal particles from 6.0 to around pH 5.0, thus preventing Zein from accumulating near its native IEP (pH 6.2) and is the most commonly used stabilizer for Zein nanoparticles (108, 109). In addition, the addition of sodium caseinate can provide spatial stability for Zein nanoparticles and improve the poor redispersibility of Zein after lyophilization (110). Some studies suggest that sodium caseinate modifies Zein nanoparticles by electrostatic adsorption (111). Patel et al. studied the FT-IR spectra of Zein nanoparticles and caseinate stabilized Zein nanoparticles and found that the FT-IR spectra of Zein nanoparticles modified with sodium caseinate did not show significant peak shift or the appearance of new peaks. Meanwhile, the DSC thermograms of Zein colloidal particles and caseinate stabilized Zein colloidal particles demonstrated the absence of any chemical interaction between Zein and caseinates (109). Chang et al. used the pH-driven method to prepare Zein/caseinate/pectin composite nanoparticles with particle size of less than 200 nm, narrow distribution, spherical distribution, and strong negative charge. And Zein nanoparticles modified with sodium caseinate have good redispersability (112). Huang et al. verified the stability of Curcumin-loaded Zein/sodium caseinate-alginate nanoparticles by dispersing the nanoparticles in a solution with a pH of 2.0–8.0 and a salt concentration of 0–1.6 M NaCl. The results show that the nanoparticles have stable anti-aggregation properties in the pH range of 2.0–7.3 and at a high salt concentration of 1.6 M (113). Mohamed et al. designed and prepared Zein/sodium caseinate nanoparticles encapsulated with celecoxib (CXB) and prodigiosin (PDG) for the treatment of triple-negative breast cancer. The increased cytotoxicity of nanoparticles may be related to the nanoparticles stability and the cell absorption capacity of Zein nanoparticles enhanced by the addition of sodium caseinate (114).

### 4.2 Chitosan

Chitosan is a natural linear polysaccharide cationic and hydrophilic polymer, obtained by alkaline hydrolysis of chitin, which is not only non-toxic but also has good biocompatibility (115). Recent studies have shown that the embedding material of Zein nanoparticles without any modification is easy to release quickly in a short time (116, 117). To overcome this problem, polysaccharides were used as colloidal stabilizers to coat Zein nanoparticles (118, 119). Chitosan has rich hydroxyl (−OH) and amine (−NH_2_) functional groups, which can be used to react with crosslinking agents for *in situ* chemical crosslinking (120). Hence, chitosan as a cationic polysaccharide has attracted much attention due to its great potential in the development of Zein nanoparticles (121). Zhang et al. (122) designed liver-specific targeted nanoparticles naringenin-Zein-sodium caseinate-chitosan nanoparticles to improve the stability of Zein nanoparticles and improve the lipid-lowering activity of Naringenin. Cai et al. (123) found that chitosan modified Zein nanoparticles could achieve sustained antibacterial effect. Liu et al. (124) prepared chitosan modified Zein nanoparticles with a particle size of about 138 nm, which effectively improved the stability of Zein nanoparticles at high ionic strength, and effectively improved the thermal stability and light stability of curcumin. Li et al. (125) found that modifying Zein nanoparticles with Chitosan can not only improve the stability of the nanoparticles, but also make the nanoparticles have excellent antibacterial effect.

### 4.3 Lactoferrin

Lactoferrin is a nutrient that is typically found in mammalian milk and has antibacterial and antiviral effects (126). Lactoferrin is thought to exert its primary biological activity after interacting with receptors on target cells. Lactoferrin receptors include CD14 (127), intestinal epithelial cells and lymphocytes (128), and LDL receptor-related protein-1 (LRP-1/CD91, 129). Importantly, lactoferrin can also bind to heparan sulfate proteoglycans (HSPGs) on the cell surface (130). This can be used in targeting strategies where overexpressed receptors on the cell surface favor receptor-mediated endocytosis of nanoparticles, leading to enhanced substance delivery (131). In addition, lactoferrin can be used as a stabilizer to improve the instability of Zein nanoparticles under high salt conditions. Chen et al. prepared glycosylated lactoferrin by Maillard reaction as a stabilizer, established Zein/glycosylated LF nanoparticles and successfully encapsulated 7, 8-dihydroxyflavone (7, 8-DHF). Meanwhile, the bioaccessibility of DHF-Zein/LF was three times higher than free 7, 8-DHF through *in vitro* simulation of gastrointestinal digestion experiment (132). Sarah et al. designed active targeting nanoparticles based on the anticancer activity of lactoferrin itself and the high expression of lactoferrin receptor (LDL receptor) in breast cancer cells, and the study showed that Lactoferrin modification may enhance the targeting and internalization behavior of nanocarriers entering cancer cells (133). Wang et al. (134) found that lactoferrin can promote the absorption of NPs by intestinal epithelial cells to improve the brain permeability of CF_3_CN. According to these literatures, lactoferrin mainly modifies Zein nanoparticles by forming hydrogen bonds.

### 4.4 Folate

Folate, also known as vitamin B_9_, plays a crucial role in one-carbon transfer reactions, cell division, growth, and survival, especially in rapidly dividing cells (135, 136). Folate receptor (FR) is a 38 kDa glycosylphosphatidylinositol-anchored that binds to the vitamin folate with high affinity (137). Folate receptors are most widely expressed at very low levels in normal tissue, but it is overexpressed in many cancers, including stomach, ovarian, and breast cancers (138, 139). Many studies have shown that folate increases tumor accumulation of nanoparticles. And folate modified nanoparticles show effective targeting ability in tumor diagnosis and therapy (140–142). The methods of folic acid modification of Zein nanoparticles mainly include directly forming amide bond with Zein, forming amide bond with other compounds to modify Zein nanoparticles and some modifications without chemical reaction. For instance, Wu et al. (143) used an amide coupling agent to activate the carboxyl group of folate, which combined with the amino group of Zein to form an amide bond, and used this material to prepare targeted nanoparticles. Zar et al. (144) first reacted folic acid with PEG to obtain FA-PEG-COOH and then reacted this substance with Zein to prepare nanoparticles. In addition, Wang et al. (145) and Wusigale et al. (146) did not use chemical coupling to design folate-modified Zein targeting nanoparticles. The variety of modification methods of folic acid make it widely used in the modification of targeted nanoparticles. Folic acid-modified Zein nanoparticles FA-NP-DOX were prepared by Hou et al. The results *in vivo* pharmacokinetic studies showed that DOX was cleared from the circulation after 4 h, DOX from NP-DOX was completely released and cleared after 7 h, while DOX from FA-NP-DOX remained in the circulation after 24 h. This demonstrated that FA-NP-DOX could effectively prolong the release of antitumor drugs. Besides, *in vivo* anticancer studies demonstrated that FA-NP-DOX significantly inhibited tumor growth (147). Research has shown that folate receptor levels are significantly higher in inflammatory sites than in normal tissues. Wu et al. modified Zein nanoparticles with folate and observed the distribution of nanoparticles in colonic inflammatory tissues by using frozen section technology combined with laser confocal microscopy. The results showed that the fluorescence intensity of Zein nanoparticles modified with folate was stronger than that of unmodified Zein nanoparticles, which demonstrated that folate modification could target and enrich nanoparticles at the site of inflammation (148).

### 4.5 Hyaluronic acid

Hyaluronic acid is an anionic non-sulfated glycosaminoglycan, and it is widely distributed in connective tissue and epithelial tissue (149). Hyaluronic acid is essential component of extracellular matrix (150, 151). Meanwhile, it can bind specifically to receptors such as CD44, GHAP (glionic acid binding protein), and TSG6 (TNF-stimulating gene 6, 152). Current studies have shown that CD44 is of major significance (153). CD44 is a non-kinase transmembrane glycoprotein and is overexpressed in several cell types, including cancer stem cells (154). Hyaluronic acid is the main ligand of CD44, which binds to CD44 and activates CD44, leading to cell proliferation, adhesion, migration, and invasion (155, 156). CD44 may be a molecular target for cancer therapy and an important prognostic marker (157). Hydrogen bonding, electrostatic and hydrophobic interactions between Zein and hyaluronic acid molecules may be responsible for the formation of stable complexes in the nanoparticles (158). Seok et al. (159) successfully developed hyaluronic acid cross-linked Zein nanoparticles to deliver curcumin to CD44-expressing cancer cells. Zhang et al. (160) designed Zein nanoparticles loaded with the anticancer drug Honokiol (HNK) and modified them with hyaluronic acid. HA-Zein-HNK could be delivered in a targeted manner to improve the therapeutic efficacy in breast cancer treatment.

### 4.6 Intelligent nanoparticles

#### 4.6.1 pH-responsive nanoparticles

The rapid growth of tumor tissue can lead to hypoxia (161). Therefore, glycolysis occurs at the tumor site to produce acidic metabolites, resulting in a tumor site pH lower than the physiological pH value (162). Kaushik et al. designed pH-dependent hydrogel-modified Zein nanoparticles, which could effectively release adriamycin into the cellular acidic environment of HeLa cells. In addition to their application in cancer targeting, the research on targeted delivery of pH-response nanoparticles in the gastrointestinal tract is also of great significance. Li et al. proposed glycyrrhizic acid (GA) as pH-responsive substances to functionalize curcumin-loaded Zein nanoparticles. As the pH increases from 3 to 7. The release of GA from the surface of nanoparticles leads to a change in the stability of Zein nanoparticles, and the curcumin is released from the nanoparticles, achieving the effect of intelligent control of drug release (163). At the same time, the PH-responsive Zein nanoparticles have been applied in the treatment of diabetes (164), food preservation (19, 165), and improving the water dispersity of pesticides (166).

#### 4.6.2 Magnetically responsive nanoparticles

Magnetic nanoparticles (MNP) are prepared by metal materials or magnetic nanoparticle composite materials. Under the guidance of magnetic field *in vitro*, the magnetically responsive nanoparticles can accumulate at the target site (167). Nanoparticles with magnetic response usually consist of two structures, the first one is modification on the surface of magnetic nanoparticles and the second one is encapsulation of magnetic material in nanoparticles (168, 169). Pang et al. prepared nanoparticles from superparamagnetic iron oxide nanoparticles (SPIONs) and gefitinib (GEF) encapsulated in folate-conjugated Zein (Fa-Zein). The experimental results showed that the uptake of GEF into A549 cells was facilitated by utilizing the magnetic response property of SPIONs and the active targeting of folate, which enhanced the toxicity of GEF to A549 cells (170). In addition to the research of cancer-targeting nanoparticles, magnetically responsive Zein nanoparticles are also of great significance in environmental protection and food detection (171, 172).

#### 4.6.3 Photo-responsive nanoparticles

Currently, photodynamic/photothermal therapy of photosensitizers is currently undergoing intensive preclinical and clinical studies (173). Lee et al. used the Zein-phosphatidylcholine hybrid nanoparticles (Z/PC-NP) as drug carrier to prepare light-sensitive nanoparticles loaded with near-infrared dye indocyanine green (ICG). ICG encapsulated in Z/PC-NP is twice as phototoxic to cancer cells as PC-NP (174). Abdelsalam et al. (175) designed light-responsive nanoparticles of Zein modified with the natural photosensitizer hypericin and investigated its active targeting of HepG2 cells by apoptosis assay, the results showed that the apoptosis was more obvious in hypericin-modified Zein nanoparticles group.

## 5 Conclusion and outlook

Zein is a plant protein with unique solubility, biocompatibility and low immunogenicity. In recent years, Zein-based nano-delivery system has been widely used in pharmaceutical, food, agriculture, environmental protection and other fields. Emerging technologies for the preparation of Zein-based nano-delivery systems play an important role in the preparation of nanoparticles that are stable, actively targeted, intelligently responsive, and multifunctional hybrids. This paper reviews the current status of research on different preparation methods and modifications of Zein-based nano-delivery systems based on the basic properties of Zein. The low immunogenicity and good biocompatibility of Zein are essential parameters for its wide range of applications in biomedical, pharmaceutical and other fields. Evidence in the literatures suggest that Zein nanoparticles with different particle sizes and size distributions can be produced by using different preparation methods. Although Zein nanoparticles with narrow particle size distribution, higher encapsulation efficiency and drug loading capacity can be obtained by different preparation methods, Zein-based nano-delivery systems are widely known for their poor colloidal stability. The selection of different additives to enhance its stability has become a hot research topic.

The development trend of Zein-based nano-delivery system in the future is mainly the design of active targeting and the expansion of industrialization. Firstly, although unmodified Zein nanoparticles can improve the problem of low drug solubility and poor stability, their entrapment efficiency at the targeting site is very limited. The design of Zein nanoparticles with active targeting function can effectively improve the enrichment efficiency of drugs and improve drug utilization efficiency. Secondly, Zein is easy to modify and has favorable biocompatibility and degradability, but the effect of biodegradation of modified Zein derivatives is less studied at present. The last is to realize the expansion of Zein nanoparticle preparation technology from laboratory to industrialization. With the rapid development of nanoparticle preparation technology, small-scale preparation of Zein nanoparticles in the laboratory has achieved satisfactory stability and reproducibility, but how to industrialize its large-scale production has become a major obstacle limiting the application of Zein nanoparticles at present. Therefore, the design and development of software, which can predict the parameters of nanoparticles may be a solution to solve the difficulties in the industrialization of nanoparticle preparation technology. The fact that Zein is cheap and easily available as a biomaterial and the presence of different groups in its polymer chain (amine, amide, hydroxyl, carboxylate, and phenol) gives it a wide range of modification possibilities. In addition, the material can be prepared into drug delivery systems of different sizes and shapes, which has a very high potential for applications.

## Author contributions

XL: Writing – original draft, Writing – review & editing. MZ: Writing – review & editing. XZ: Writing – review & editing. MW: Writing – review & editing. AC: Writing – review & editing. BX: Writing – review & editing. JY: Supervision, Writing – review & editing. HL: Writing – review & editing, Supervision.
